# Evidence-Based Health: Necessary But Not Sufficient

**Published:** 2011-12-15

**Authors:** Elizabeth Herman, Paul Garbe

**Affiliations:** Air Pollution and Respiratory Health Branch, National Center for Environmental Health, Centers for Disease Control and Prevention, Atlanta, Georgia; Air Pollution and Respiratory Health Branch, National Center for Environmental Health, Centers for Disease Control and Prevention, Atlanta, Georgia

## To the Editor:

As public health practitioners from the National Asthma Control Program (NACP) of the Centers for Disease Control and Prevention, we read the essay "From Evidence-Based Medicine to Evidence-Based Health: the Example of Asthma" (1) with great interest. We agree with the authors' hypothesis that evidence-based clinical medicine must be supplemented by asthma self-management support "that extends beyond the clinic" and "by interventions that change elements of the environment in which patients live." As the authors note, this concept is not new. Indeed, it underlies the Expert Panel Report 3: Guidelines for the Diagnosis and Management of Asthma (2).

A recent article (3) describes remarkable progress not only in understanding the pathophysiology of asthma and in producing new medications for its control but also in a public health response "to support patient- and community-level interventions and to assess the effect of the environment on asthma." The NACP has greatly expanded population-level asthma surveillance of asthma prevalence, illness, and death (4). The NACP also supports 34 states, Washington, DC, and Puerto Rico "to build and sustain programs that translate evidence-based practice into interventions." Furthermore, the Task Force on Community Preventive Services recently conducted and published a systematic review of the effectiveness of home-based, multi-trigger, multicomponent interventions in improving asthma control (5). The NACP is working through state asthma programs to implement those interventions.

Much work remains to be done to achieve evidence-based health (as defined by Moskowitz and Bodenheimer), particularly among racial/ethnic minorities, who have a disparately high prevalence of and morbidity from asthma. The authors note 3 necessary actions: linking clinical teams with community resources to address asthma triggers in housing, advocating for better housing and cleaner air, and convincing insurers to reimburse for essential educational and community health services. We suggest that these actions, although necessary, are not sufficient to decrease the burden of asthma at a population level.

Although sufficient evidence exists to direct the clinical management of asthma, there is an urgent need to expand the evidence for cost-effective ways to implement medical and behavioral interventions on a large scale and among diverse settings and communities. Moskowitz and Bodenheimer cite reports of successful asthma interventions in several communities. These interventions, although key demonstration projects, are the equivalent of clinical case studies. Just as experts would not base clinical guidelines on case studies, program planners and policy makers should not base decisions about community health interventions on a few demonstration projects.

No national system exists to direct program implementation questions back to an organized research effort and address them systematically. Both the research to determine the most cost-effective strategies for ensuring that evidence-based treatments reach the populations most in need and the programs charged with implementing those strategies are grossly underfunded. As Steven Woolf argues (6), the vastly disproportionate funding of research to develop new treatments and procedures over research to deliver existing ones more effectively and equitably does not advance the public's health; indeed "the current policy of spending [only] 1.5% of research dollars on health services research is probably costing lives."

Thus, we agree with Moskowitz and Bodenheimer that implementing evidence-based health "is essential to reduce the burden of asthma and other chronic diseases and to help control the associated costs to society." We add, however, that implementation is not a matter of "just doing it." Implementation is a science that requires a systematic cycle of hypothesis formation, testing, analysis, feedback, and dissemination. It should be valued and funded at a level that reflects its potential for improving the public's health.
